# Supplementary material for: Artificial intelligence that determines the clinical significance of capsule endoscopy images can increase the efficiency of reading
Source: PLoS One. 2020 Oct 29;15(10):e0241474. doi: 10.1371/journal.pone.0241474 (PMC7595411; doi:10.1371/journal.pone.0241474)
Supplement: Attachment — Submitted filename: Response to Reviewers.docx [file pone.0241474.s001.docx]

Date: July. 27. 2020

Dilbag Singh

Academic Editor

PLOS ONE

Dear Editor;

Thank you for inviting us to submit a revised draft of our manuscript entitled, “Artificial intelligence that determines the clinical significance of capsule endoscopy images can increase the efficiency of reading” to PLOS ONE. We also appreciate the time and effort you and each of the reviewers have dedicated to providing insightful feedback on ways to strengthen our paper. Thus, it is with great pleasure that we resubmit our article for further consideration. We have incorporated changes that reflect the detailed suggestions you have graciously provided. We also hope that our edits and the responses we provide below satisfactorily address all the issues and concerns you and the reviewers have noted.

Including this rebuttal letter, I attached 3 separate files labeled ‘Response to Reviewers’, ‘Revised Manuscript with Track Changes', and ‘Manuscript’.

Please check the answers below. Each reviewer’s comment was replied with sentences in bold text.

1. Please ensure that your manuscript meets PLOS ONE's style requirements, including those for file naming. The PLOS ONE style templates can be found at

https://journals.plos.org/plosone/s/file?id=wjVg/PLOSOne_formatting_sample_main_body.pdf and

https://journals.plos.org/plosone/s/file?id=ba62/PLOSOne_formatting_sample_title_authors_affiliations.pdf

**In the revised paper, we corrected the organization according to the instructions of formatting.**

2. Please amend either the abstract on the online submission form (via Edit Submission) or the abstract in the manuscript so that they are identical.

**We matched the online submission form of abstract and the modified abstract following the reviewer’s recommendation when we submitted the revised article.**

3. We note that Figure 2-3 in your submission contain satellite images which may be copyrighted. All PLOS content is published under the Creative Commons Attribution License (CC BY 4.0), which means that the manuscript, images, and Supporting Information files will be freely available online, and any third party is permitted to access, download, copy, distribute, and use these materials in any way, even commercially, with proper attribution. For these reasons, we cannot publish previously copyrighted maps or satellite images created using proprietary data, such as Google software (Google Maps, Street View, and Earth). For more information, see our copyright guidelines: http://journals.plos.org/plosone/s/licenses-and-copyright.

**Thank you for the considerable review. However, the images of this article do not contain any copyrighted satellite information. If you point out** **the colorized features of images 2 and 3, they were created with open-source technology of the “Class activation mapping”. It was mentioned as a reference in the manuscript. You can also find the information** **in the website, “https://github.com/zhoubolei/CAM”.**

**Reviewers' comments:**

Reviewer #1: After initial screening I have observed following issues:

1. Authors should add more comparative analyses,

**As you recommended, we added per lesion analysis results of lesion detection rates and reading time of reviewers. We also found a correlation between the total number of extracted images and the number of images exceeding the significance threshold. The statistical correlations and interpretations were discussed in the Results section and the Discussion section.**

2. It is advised to explain the equations present in the paper in more efficient manner.

**We explained the learning, validation and test process of AI development with more detailed parameters. We also proved this by comparing the AUC values that random selection of images to construct each dataset was performed in an appropriate way.**

3. Some confusing and long lines are there, authors should adjust these lines.

**We corrected some confusing lines and attached the certificate of English proofreading by a professional company.**

4. The literature can be improved by adding some recent plos one papers and also: Efficient Prediction of Drug-drug interaction using Deep Learning Models, Imputing missing values using cumulative linear regression, Visibility improvement and mass segmentation of mammogram images using quantile separated histogram equalisation with local contrast enhancement, Classification of COVID-19 patients from chest CT images using multi-objective differential evolution--based convolutional neural networks, Deep learning approach for microarray cancer data classification, Deep Transfer Learning based Classification Model for COVID-19 Disease.

**Unfortunately, the papers presented as examples are not suitable for use as references of this article. However, PLOS ONE has many valuable references about AI in medical imaging diagnosis and 3 of them were cited in the revised paper.**

5. Conclusion can be improved further by presenting more statistical computed values.

**As you recommended, we empowered discussion and conclusions with statistical values.**

Reviewer #2:

1. Author should reconsider the title. It is very confusing.

**As you recommended, we modified the title with more clarified words. We also attached the certificate of English proofreading by a professional company.**

2. Abstract is too long. Authors need to be rewrite it for better understanding.

**We shortened the abstract using 215 words. The re-written abstract contains all the abstract contents required by the author's guidelines.**

3. It is advised that ROC curve that is Figure 4 must be reevaluated by considering ROC analysis of competitive techniques.

**We explained the learning, validation and test process of AI development with more detailed parameters. We also proved this by comparing the AUC values that random selection of images to construct each dataset was performed in an appropriate way.**

4. Figure 5 is confusing, so authors should provide more description about this figure.

**We added per lesion analysis results of lesion detection rates and reading time of reviewers. The statistical correlations and interpretations were discussed in the Results section and the Discussion section. In the revised papers, previously not mentioned numbers are introduced. We are sure these works will improve your understanding of the graph.**

5. It is advised to compare with more state-of-art techniques for comparative analyses.

**Although related to the recent AI research, this study mainly covers the field of clinical medicine. The primary outcome of this study is to see the clinical effectiveness of AI, not to prove the technological performance of AI. Analysis of whether the detection of clinical lesions could be improved with the help of AI was properly assessed using classical techniques of statistics. They effectively demonstrated the difference between the two reviewer groups. In addition, the AI technology used to assist the CE reading is also selected from the recently developed high-performance networks. As reported in the 11^th^ reference, the Inception-ResNet-V2 shows the third highest accuracy among various deep learning models. Because the other two highest models have much more parameters, we select Inception-ResNet-V2 to consider accuracy and training/inference time simultaneously.**

6. Parameter setting of the proposed model should be presented in tabular form.

**In the revised paper, we presented the detail of parameters in Table 1.**

7. There are some typos and grammatical mistakes present in the manuscript.

**We corrected the typos and grammatical mistakes. We also attached the certificate of English proofreading by a professional company**

8. Paper organization is missing at the end of introduction

**In the revised paper, we corrected the paper organization according to the instructions of formatting.**

9. Headings and sub-headings have not been marked

**In the revised paper, we marked the headings and sub-headings according to the instructions of formatting.**

10. It is advisable to authors consider following useful references

Fusion of medical images using deep belief networks, Classification of COVID-19 patients from chest CT images using multi-objective differential evolution–based convolutional neural networks, Efficient prediction of drug–drug interaction using deep learning models, Deep Transfer Learning based Classification Model for COVID-19 Disease, Multi-objective differential evolution based random forest for e-health applications

**Unfortunately, the papers presented as examples are not suitable for use as references of this article. However, PLOS ONE has many valuable references about AI in medical imaging diagnosis and 3 of them were cited in the revised paper.**

Reviewer #3: Paper can be improved further

1. Add suitable research motivation

**We modified the introduction section. We emphasized the distinction of our study and clarified the goals. We presented a practical comparison method considering the reality in capsule endoscope reading and suggested that the statistical interpretation through this is our goal.**

2. Add more relevant references from plos one.

**PLOS ONE has many valuable references about AI in medical imaging diagnosis and 3 of them were cited in the revised paper.**

3. Equations should be explained carefully

**We explained the learning, validation and test process of AI development with more detailed parameters. We also proved this by comparing the AUC values that random selection of images to construct each dataset was performed in an appropriate way.**

4. More comparative analysis are required

**As you recommended, we added per lesion analysis results of lesion detection rates and reading time of reviewers. We also found a correlation between the total number of extracted images and the number of images exceeding the significance threshold. The statistical correlations and interpretations were discussed in the Results section and the Discussion section.**

5. Add statistical analysis in conclusion or discussion session

**As you recommended, we empowered discussion and conclusions with mentioning the statistical analysis.**

I hope the above answers provided sufficient explanations for each comment. Again, thank you for giving us the opportunity to strengthen our manuscript with your valuable comments and queries. We have worked hard to incorporate your feedback and hope that these revisions persuade you to accept our submission.

Sincerely,

Yun Jeong Lim, M.D.

Department of Internal Medicine, Dongguk University Ilsan Hospital

27 Dongguk-ro, Ilsan-dong-gu, Goyang 10326, Korea

Tel: +82-31-961-7133

Fax: +82-31-961-7730

E-mail: drlimyj@gmail.com
